# DNA barcoding of Naididae (Annelida, Oligochaeta), based on cytochrome C oxidase gene and ITS2 region in China

**DOI:** 10.3897/BDJ.9.e73556

**Published:** 2021-12-14

**Authors:** Tingting Zhou, Wei Jiang, Hongzhu Wang, Yongde Cui

**Affiliations:** 1 State Key Laboratory of Freshwater Ecology and Biotechnology, Institute of Hydrobiology, Chinese Academy of Sciences, Wuhan, China State Key Laboratory of Freshwater Ecology and Biotechnology, Institute of Hydrobiology, Chinese Academy of Sciences Wuhan China; 2 University of Chinese Academy of Sciences, Beijing, China University of Chinese Academy of Sciences Beijing China

**Keywords:** aquatic oligochaete, Naididae, DNA barcoding, COI, ITS2

## Abstract

Exploring the effectiveness of DNA barcoding in species identification is a prerequisite for biodiversity conservation and environmental monitoring. Aquatic oligochaetes could serve as excellent indicators in aquatic monitoring programmes. However, few studies have examined the effectiveness of DNA barcoding in these specific organisms. The mitochondrial cytochrome C oxidase (COI) gene of 83 specimens belonging to 40 species of 18 genera were sequenced in this study. The results showed that there was a barcode gap between species of Naididae and the intraspecific genetic distances of each species were smaller than interspecific genetic distances. The classification results of ABGD (Automatic Barcode Gap Discovery) were consistent with those of morphological identification, except for *Tubifextubifex* and *Lumbriculusvariegatus*. All species were successfully distinguished in the phylogenetic tree, based on the ITS2 region, which was coincident with the morphological result. Our results provided evidence that DNA barcoding can be used as an effective and convenient tool for species identification of the family Naididae and even for other aquatic oligochaetes.

## Introduction

The family Naididae, as the most diverse family within the class Oligochaeta, includes more than 1,100 valid species (Timm 2017). The Naididae is widely distributed in surface freshwater, groundwater and oceans around the world (Martin et al. 2007). There are nine known subfamilies of Naididae worldwide, amongst which four subfamilies are common in China, including Naidinae, Tubificinae, Rhyacodrilinae and Pristininae (Wang and Cui 2008). The Naididae include species showing different degrees of tolerance to pollution, which explains why they are used as bioindicators and not only tolerant species. Aquatic oligochaete communities are used for assessing environmental conditions (e.g. sediment and water quality) and establishing ecological diagnostics. The identification of aquatic oligochaetes, based on DNA barcoding, will greatly facilitate the development of biological monitoring (Lafont et al. 2010, Lafont et al. 2012, Vivien et al. 2020a). Recently, high-throughput and metabarcoding techniques are assisting in the development of oligochaete indices (Vivien et al. 2019, Vivien et al. 2020b).

Rapid and unambiguous identification of species is an essential prerequisite for environmental monitoring and biodiversity (Xiao et al. 2004, Cheng et al. 2011). Both experienced taxonomists with solid professional knowledge and a relatively intact biological specimen are necessary for traditional morphological identification. However, experts who engage in taxonomic research continue to decline. The main identification characteristics, based on genitalia, constitute a major obstacle to the identification of immature species. For example, the species, *Limnodrilushoffmeisteri* Claparède, 1862 and *L.claparedianus* Ratzel, 1868 can be identified only when the specimens are in a mature state (Yasuda and Okino 1988). In addition, traditional methods are still limited due to high cost and an immense amount of time. With the rapid development of molecular biology, molecular research of species identification comes into being.

DNA-based identification methods can not only quickly and accurately identify morphologically damaged specimens (Carvalho et al. 2015), but also effectively identify species at various developmental stages (Casiraghi et al. 2010). Thereinto, the cytochrome C oxidase subunit I (COI) of mitochondrial gene is an ideal gene target to be used as DNA barcode for species identification due to maternal inheritance with moderate sequence conservatism and variability (Hebert et al. 2003a, Hebert et al. 2003b, Remigio 2003). DNA barcoding through a short and standardised fragment of the COI gene to identify and classify species has been demonstrated in multiple taxa (Ball et al. 2005, Costa et al. 2007, Zhou et al. 2007, Foottit et al. 2008, Amer 2021, Kheirallah 2021, Rewicz et al. 2021). The technique allows identification of animal groups at the species level, as well as helps in the discovery of cryptic species (Ardura et al. 2010, Lin et al. 2020). For example, Achurra et al. (2011) revealed the cryptic diversity of *Troglodrilusgalarzai* on the basis of DNA barcoding. However, DNA barcoding, based on the COI gene alone, could be overestimating the number of species in the species delimitation of some groups of aquatic oligochaetes (Dasmahapatra et al. 2010, Achurra and Erséus 2013, Martinsson et al. 2013). Hence, the COI gene may be not suitable for phylogenetic analysis as a single molecular marker (Zhou et al. 2010). Besides, internal transcribed spacer 2 (ITS2) of the nuclear gene was often used to serve as a complementary strategy to COI in phylogenetic analysis (Envall et al. 2012). The effectiveness of this integrative method (COI and ITS2) has been demonstrated in the identification of various taxa of oligochaete, such as *Rhynchelmis* (Zhou et al. 2010), *Stylodrilusheringianus* (Achurra and Erséus 2013), *Rhyacodrilusfalciformis* (Martinsson et al. 2013), *Grania* (De Wit and Erséus 2010), *Enchytraeusalbidus* (Erséus et al. 2019), *Aporrectodealonga* (Martinsson et al. 2015) and so on.

Recent advances in developed countries have established a national barcode database of aquatic oligochaete (Vivien et al. 2017), which was evidenced as a useful tool in conserving biodiversity and environmental monitoring (Vivien et al. 2015). However, this knowledge is still lacking in China, despite embodied within many endemic oligochaetes (Cui and Wang 2012a, Cui and Wang 2012b, Peng et al. 2014, Cui et al. 2015, Peng et al. 2017). Considering that differences in regional species pool between different climate zone, we should not directly extrapolate the genetic information. In this case, establishing a regional database of aquatic oligochaete is necessary for future monitoring.

In this study, we sequenced the COI sequences of 83 specimens under 40 species belonging to 18 genera to study the barcoding of Naididae. A total of 75 sequences of the ITS2 region, including 28 GenBank sequences and 47 new sequences, were used as additional evidence. We aim to explore the accuracy of DNA barcoding technology for the species identification and to construct a bio-identification system for Naididae.

## Material and Methods

### Specimen collection

The specimens were collected between 2017 and 2020 in China (Suppl. material 1). The worms were preserved in 95% ethanol in the field. Back in the laboratory, specimens were identified, based on morphological characteristics, such as external morphology of the worm, chaetal features, penis sheath and so on. Then the worms were divided into two parts. The anterior part was stained with borax carmine and mounted in Canada balsam on a microscope slide as morphological evidence. The rest of the same specimen was preserved in 95% ethanol for future molecular studies. The vouchers are deposited in the Institute of Hydrobiology (IHB), Chinese Academy of Sciences (CAS), Wuhan, China.

### DNA extraction and PCR amplification

Total genomic DNA was extracted using the TIANGEN blood tissue kit, following the manufacturer’s protocol strictly (TIANGEN Blood and Tissue Handbook). Approximately 658 bp were amplified of the COI gene using universal primers, LCO1490-GGTCAACAAATCATAAAGATATTGG and HCO2198-TAAACTTCAGGGTGACCAAAAAATCA (Folmer et al. 1994, Bely and Wray 2004). The 25 μl PCR reaction mixes included 12.5 µl of Q5 Polymerase, 2.5 µl of 10 µmol/l of primer pair mix, 2 µl of DNA template and 8 µl ddH2O. The PCR procedures comprised an initial pre-denaturation step at 98°C for 30 sec, followed by 35 cycles of denaturation at 98°C for 10 sec, annealing at 45°C for 45 sec and elongation at 72°C for 45 sec, with an ultimate elongation at 72°C for 3 min. The recently designed specific primers of ITS2 (606F-GTCGATGAAGAGCGCAGCCA and 1082R-TTAGTTTCTTTTCCTCCGCTT) for aquatic oligochaete were chosen (Liu and Erséus 2017). The annealing temperature of ITS2 region was 55°C and the other conditions were the same as that of COI gene. A total of 5 µl PCR products were detected by 1% agarose gel electrophoresis and the remaining products were sent to I-congene Ltd. (Wuhan, China) for direct Sanger sequencing. Bidirectional sequencing was performed to improve sequence accuracy.

### Genetic analyses

Raw sequences were calibrated in BioEdit and assembled in SeqMan (DNASTAR). All sequences were aligned by ClustalW using MEGA5. The newly-acquired COI sequences in our study were compared to Genbank (NCBI) sequences using BLAST.

#### Sequence composition analyses

All of COI sequences were imported into MEGA5 for multi-sequence alignment and base composition, conserved sites, variable sites, parsimony informative sites and transitions/transversions were calculated, respectively.

#### Genetic distance analyses

The uncorrected pairwise genetic distances between sequences were obtained with MEGA5 (Tamura et al. 2011), including various taxonomic levels, species, genus and subfamily.

#### Automatic barcode gap discovery (ABGD) analyses

The genetic distance matrix of all Naididae specimens was calculated using the Kimura two parameter (K2P) model (Kimura 1980). The analysis of the division of taxa was carried out on the ABGD website (https://bioinfo.mnhn.fr/abi/public/abgd/abgdweb.html). The prior intra-specific divergence (P) was between 0.001 and 0.1 and the minimum relative gap width (X) was 1.0, with the remaining parameters leaving default.

#### Phylogenetic analyses

The Bayesian trees were generated using the software Phylosuite1.2.1 (Zhang et al. 2019) to provide a graphic showing the genetic divergence between species. The optimal substitution model was selected, based on the BIC standard (Kalyaanamoorthy et al. 2017), with GTR+F+I+G4 for both the gene and ITS2 region. Bayesian Inference phylogenies were inferred using MrBayes 3.2.6 (Ronquist et al. 2012) under the GTR+I+G+F model (2 parallel runs), in which the initial 25% of sampled data were discarded as burn-in. Bayesian analysis included 2 million generations and the posterior probability indicated the confidence of each clade of the phylogenetic tree. The Neighbour Joining (Saitou and Nei 1987) tree of the COI gene was established, based on the K2P model (Kimura 1980) with 1000 bootstrap replications in MEGA5 (Tamura et al. 2011). All positions containing gaps and missing data were completely deleted. The phylogenetic tree was submitted to iTOL website (https://itol.embl.de/itol.cgi) for online editing, then the tree graph was exported to be modified in Adobe Illustrator.

## Results

### Sequence composition analyses

A total of 40 species belonging to 18 genera were analysed, giving altogether 83 COI sequences. Ten of them were acquired for the first time, including *Tubifexlaxus* Peng, Wang & Cui, 2017; *T.conicus* He, Wang & Cui, 2012; *Isochaetidespalmatus* He, Cui & Wang, 2012; *Derodorsalis* Ferronière, 1899; *Haemonaiswaldvogeli* Bretscher, 1900; *Naissimplex* Piguet, 1906; *N.inflata* Liang, 1963; *N.badia* Peng, Wang & Cui, 2014; *N.longidentata* Cui, He, Peng & Wang, 2015 and *Rhyacodrilussinicus* (Chen 1940).

The aligned length for COI gene used in the study was 658 bp and the sequences were AT-biased (60.8%). There were 337 conserved sites and 321 variable sites, of which 319 were parsimony informative. The value of transitions/transversions was 1.35.

A total of 75 ITS sequences were obtained in this study, of which 47 were successfully amplified and the remaining 28 were from GenBank. Compared with the COI gene, the ITS2 region is often difficult to sequence.

### COI variation analyses

The average genetic distances between species range from 7.6% to 26.5%. Amongst them, *N.longidentata* and *N.communis* had the smallest inter-specific distances, while *Limnodrilusgrandisetosus* and *Lumbriculusvariegatus* had the largest.

**Figure 1.**
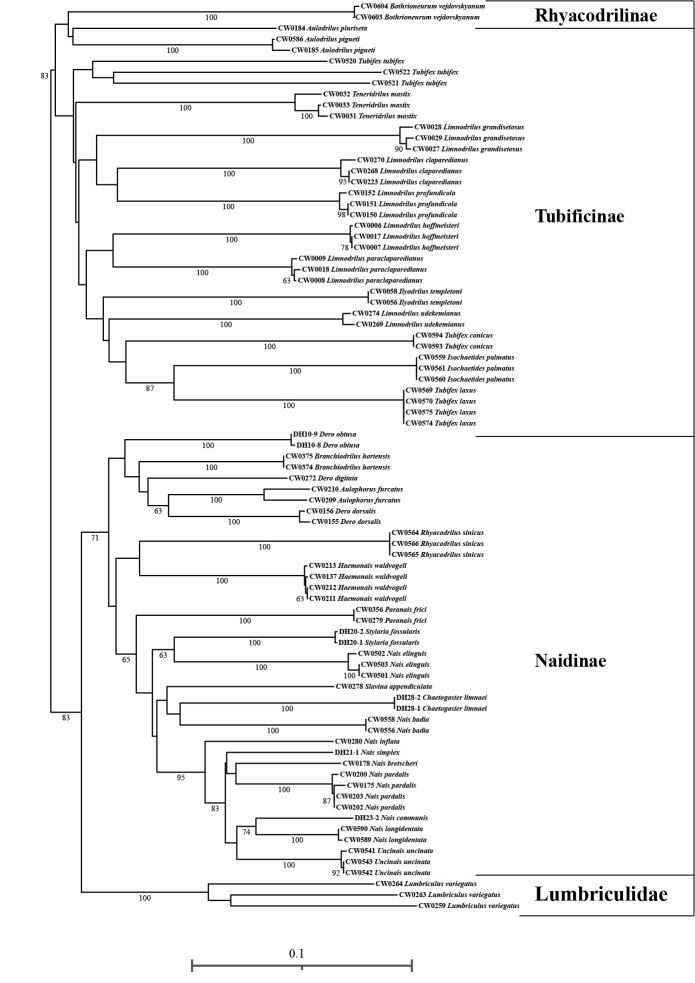
Phylogenetic tree of the COI gene, based on Neighbour-Joining analysis of Naididae. Bootstrap support > 60 are indicated.

The mean intra-specific genetic distance was 0.0-3.9% and *Aulophorusfurcatus* was maximum. The phylogenetic tree of COI sequences, based on the neighbour-joining method (NJ) was established to visualise the genetic distances (Fig. 1). The intra-specific and inter-specific genetic distances of various species of Naididae are shown in Suppl. material 2.

Overall, our results showed that there was more variation amongst species than intraspecific differences.

### ABGD analyses

**Figure 2.**
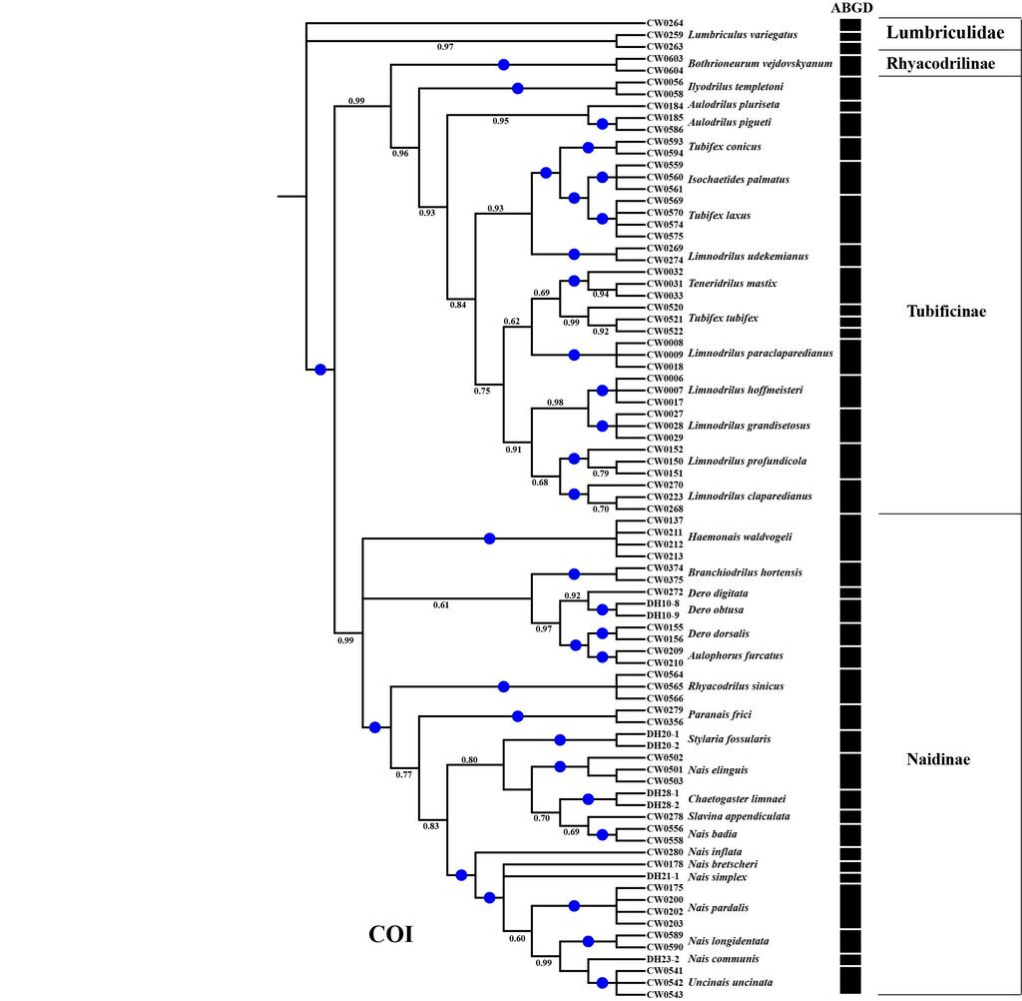
Bayesian analysis of Naididae, based on the COI gene (Automatic partition results of ABGD). BI posterior probabilities > 0.60 are indicated and the circles represent 1.00.

The partition results, based on the ABGD method, included recursive partition and initial partition and the latter was relatively stable. The COI sequences were grouped into 40 taxa (Fig. 2). Compared with the morphological results, we found that *T.tubifex* were sorted into three groups and *Lumbriculusvariegatus* were also divided into three groups, as well as the remaining 77 specimens being separated into 34 groups, which were consistent with the morphological results.

### Phylogenetic analyses

**Figure 3.**
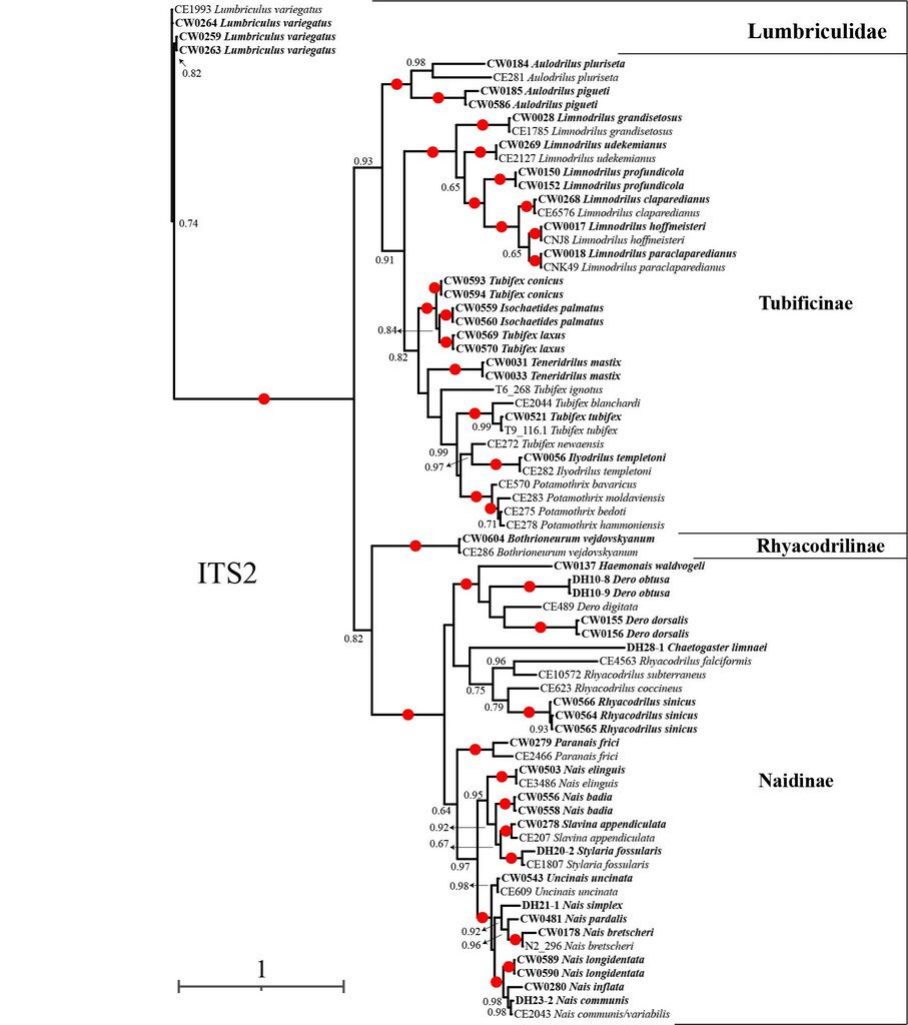
Phylogenetic tree of Naididae, based on the ITS2 region. BI posterior probabilities > 0.60 are indicated and the circles represent 1.00.

The phylogenetic analysis of family Naididae, based on the ITS2 region was constructed by Bayesian Inference (Fig. 3). Each species was well separated from each other. All Naididae sequences clustered together in the tree, based on the ITS2 region. The Naididae was monophyletic, which mainly consisted of three parts: Tubificinae, Rhyacodrilinae and Naidinae. Within the subfamily Tubificinae, the genera *Aulodrilus*, *Limnodrilus*, *Ilyodrilus* and *Potamothrix* were monophyletic and well supported (BI 1.00). There were two genera in the subfamily Rhyacodrilinae, namely, *Bothrioneurum* and *Rhyacodrilus*. However, these two taxa did not branch together and the genus *Rhyacodrilus* branched with the other Naidinae species. Hence, the subfamilies Rhyacodrilinae and Naidinae appeared as polyphyletic. The genus *Nais* was non-monophyletic, founded in two clades (BI 0.97). One clade comprised *N.badia*, *N.elinguis*, *Stylariafossularis* and *Slavinaappendiculata* and the other consisted of *N.simplex*, *N.longidentata*, *N.communis*, *N.inflata*, *N.pardalis*, *N.bretcheri* and *Uncinaisuncinata*. The genus *Tubifex* was not monophyletic.

## Discussion

The core principle of DNA barcoding is that inter-specific genetic distance is greater than intra-species differences (Hebert et al. 2003a). The genetic distance increases with the distance of the relationship. In this study, there was more variation amongst species of the same subfamily than amongst congeneric species. The genetic divergence amongst species of the same genus was greater than that of conspecific individuals. Overall, the distance between species was greater than the intra-species distance and the distance within species was generally less than 2%. Our result demonstrated the existence of cryptic species in *T.tubifex*. Sturmbauer et al. (1999) and Beauchamp et al. (2001) studied the molecular phylogeny of *T.tubifex* in northern Europe and North America, respectively and found that the maximum genetic distance of the mitochondrial 16S gene was greater than 10%. The 16S gene is more conserved than the COI gene and this value revealed a large difference within *T.tubifex*, which proved the existence of cryptic species. Genetic variation of the COI gene from Europe and North America was researched in *L.variegatus* by Gustafsson et al. (2009). *Lumbriculusvariegatus* was found to comprise two distinct clades (I and II) and the COI genetic distance was up to 17.7% between I and II. Gustafsson et al. considered that two clades were separate species. There was also a rich intra-specific diversity in *Aulodriluspluriseta*. The ITS2 genetic distance of the two specimens (CW0184: China, CE281: Estonia) of *A.pluriseta* is 17.0%. This value was far beyond the threshold of intraspecific difference, indicating the existence of cryptic species in *A.pluriseta*. The genetic distance between *A.pluriseta* and *A.japonicus* was 17.8%, which showed that they were not the same species and the same was true for morphological characteristics. There are one to several lateral teeth on bifid chaeta in *A.japonicus*, whereas they are absent in *A.pluriseta* (Finogenova and Arkhipova 1994).

Sequences can be divided quickly and effectively by the ABGD method according to the principle of "DNA barcode gap" (Puillandre et al. 2011). The result of initial partition is more stable than recursive partition, which has been confirmed by related studies (Puillandre et al. 2011, Ratnasingham and Hebert 2013). In this study, all taxa were divided into 40 groups using the ABGD method, which was consistent with the results of morphology (40 species). The existence of cryptic species of *T.tubifex* and *L.variegatus* is well-known. In addition, the COI gene, with higher evolutionary rate, is generally not enough to assess species boundaries alone (Achurra and Erséus 2013). Hence, the ITS2 region was also used to increase reliability in our study. The ABGD delimitation model provided a consistent result with morphological observations basically.

The phylogeny revealed that Naidinae and Tubificinae were monophyletic, whereas Rhyacodrilinae was not. This was supported by recent research (Erséus et al. 2020). Findings revealed a tropical freshwater origin of Naidinae, the phylogenetic relationships using combined markers by Bayesian Inference supported that *Nais* was paraphyletic and *Uncinais* was included in it (Erséus et al. 2017). *Naisbadia*, endemic to Tibet, was sister to the clade consisting of *Stylariafossularis* and *Slavinaappendicula*. We suspect this is because of pigments in the anterior segments of *N.badia* and *S.fossularis*. The genus *Tubifex* was not recovered herein as monophyletic. Many works have revealed that there are cryptic species within *T.tubifex* (Sturmbauer et al. 1999). In the ITS2 tree, *Tubifexconicus* and *T.laxus* were clustered together with *Isochaetidespalmatus*, but not with *T.tubifex*. Therefore, the taxonomic status of *T.conicus* and *T.laxus* still need to be determined in the future. *Tubifextubifex* and *T.blanchardi* are sister species. Chapman and Brinkhurst (1987) suggested that the hair chaetae of *T.tubifex* would be induced by the environment to disappear and become *T.blanchardi*, which were actually the same species. Crottini et al. (2008) suggested the variation of *T.blanchardi* and *T.tubifex* in the Lambro River, based on the 16S gene and pointed out that *T.blanchardi* and *T.tubifex* were two independent lineages. Marotta et al. (2009) re-described the morphological characteristics of *T.blanchardi* and found the differences of morphology with *T.tubifex*, thus finally confirming that *T.blanchardi* and *T.tubifex* are two separate species.

## Conclusions

The study represents the first DNA barcoding study of the aquatic oligochaete in China, including endemic species and common species. Our findings detected that DNA barcoding, based on the COI gene is practicable and effective in identifying aquatic oligochaetes; however, combining ITS2 would provide more information. The ITS2 region can be used to help build a barcode database (for delimitation of species), but not for biomonitoring. The taxonomic status of some species needs to be confirmed through comprehensive taxonomic research, by expanding the sampling range and increasing the number of specimens. The barcodes of oligochaete species need to be continuously studied to establish a native database so as to lay a solid foundation for the biological research of aquatic oligochaete.

## Supplementary Material

747C3548-B270-5053-92EC-71C46EEC96DA 10.3897/BDJ.9.e73556.suppl1 Supplementary material 1 Collection information of specimens of Naididae. Data type Collection information Brief description Collection information of specimens of Naididae. The sequences from China are shown in bold. Missing data are marked with “-”. The remaining sequences are downloaded from Genbank. File: oo_621553.docx https://binary.pensoft.net/file/621553 Tingting Zhou, Wei Jiang, Hongzhu Wang and Yongde Cu

FDFBE233-7176-50FC-A53B-58299E634925 10.3897/BDJ.9.e73556.suppl2 Supplementary material 2 Uncorrected pairwise genetic distance of COI gene for species of the family Naididae. Data type Uncorrected pairwise genetic distance Brief description Uncorrected pairwise genetic distance of COI gene for species of the family Naididae. Intra-specific distances are given as maximum distance, and intra-specific as minimum distance. Missing data are marked with “-”. File: oo_621524.xlsx https://binary.pensoft.net/file/621524 Tingting Zhou, Wei Jiang, Hongzhu Wang and Yongde Cui
