# Supplementary material for: DNA barcoding of Naididae (Annelida, Oligochaeta), based on cytochrome C oxidase gene and ITS2 region in China
Source: Biodivers Data J. 2021 Dec 14;9:e73556. doi: 10.3897/BDJ.9.e73556 (PMC8692306; doi:10.3897/BDJ.9.e73556)
Supplement: Supplementary material 1 — Collection information of specimens of Naididae. [file bdj-09-e73556-s001.docx]

Collection information of specimens of Naididae. The sequences from China are shown in bold. Missing data are marked with “-”. The remaining sequences are downloaded from Genbank.

| **Species** | **Specimen ID** | **COI** | **ITS2** | **Collection site** | **Longitude** | **Latitude** | **Collection date** |
| --- | --- | --- | --- | --- | --- | --- | --- |
| *Limnodrilus hoffmeisteri* | CW0006 | **MT450679** | - | Donghu Lake, Hubei, China | 114.352 E | 30.535 N | 2017.12.02 |
| *Limnodrilus hoffmeisteri* | CW0007 | **MT450680** | - | Huaihe River, Anhui, China | 116.290 E | 32.444 N | 2017.11.23 |
| *Limnodrilus hoffmeisteri* | CW0017 | **MT450682** | **MT109343** | Taihu Lake, Jiangsu, China | 120.191 E | 31.502 N | 2018.11.30 |
| *Limnodrilus hoffmeisteri* | CNJ8 | - | KY369418 | Chanhe Rive, Shanxi, China | 108.765 E | 34.019 N | 2013.06.12 |
| *Limnodrilus claparedianus* | CW0223 | **MT450693** | - | Yixing, Jiangsu, China | 119.604 E | 31.166 N | 2019.11.16 |
| *Limnodrilus claparedianus* | CW0268 | **MT450694** | **MT451906** | Naolihe River, Heilongjiang, China | 132.375 E | 46.342 N | 2019.10.01 |
| *Limnodrilus claparedianus* | CW0270 | **MT450695** | - | Wuxinghu Lake, Heilongjiang, China | 133.009 E | 46.812 N | 2019.10.01 |
| *Limnodrilus claparedianus* | CE6576 | - | KY369385 | Åker Canal, Hildestrandsvägen, Åkersberga, Sweden | 18.276 E | 59.496 N | 2009.06.06 |
| *Limnodrilus paraclaparedianus* | CW0008 | **MT460105** | - | Donghu Lake, Hubei, China | 114.352 E | 30.535 N | 2017.12.02 |
| *Limnodrilus paraclaparedianus* | CW0009 | **MT460106** | - | Longganhu Lake, Hubei, China | 116.132 E | 29.954 N | 2017.12.11 |
| *Limnodrilus paraclaparedianus* | CW0018 | **MT460109** | **MT109344** | Taihu Lake, Jiangsu, China | 120.191 E | 31.502 N | 2018.11.30 |
| *Limnodrilus paraclaparedianus* | CNK49 | - | KY369391 | Ku Yu valley, Shaanxi, China | 109.172 E | 34.011 N | 2013.06.12 |
| *Limnodrilus udekemianus* | CW0269 | **MT460100** | **MT451902** | Wuxinghu Lake, Heilongjiang, China | 133.009 E | 46.812 N | 2019.10.01 |
| *Limnodrilus udekemianus* | CW0274 | **MT460101** | - | Wuxinghu Lake, Heilongjiang, China | 133.007 E | 46.812 N | 2019.10.01 |
| *Limnodrilus udekemianus* | CE2127 | - | KY637023 | Univ Osnabrück, lab culture at Zool Dep, Germany Osnabrück | 8.033 E | 52.283 N | 2006.11.16 |
| *Limnodrilus grandisetosus* | CW0027 | **MT457606** | - | Taihu Lake, Jiangsu, China | 120.152 E | 31.403 N | 2019.02.23 |
| *Limnodrilus grandisetosus* | CW0028 | **MT457607** | **MT109348** | Taihu Lake, Jiangsu, China | 119.988 E | 31.035 N | 2019.02.24 |
| *Limnodrilus grandisetosus* | CW0029 | **MT457608** | - | Taihu Lake, Jiangsu, China | 119.988 E | 31.035 N | 2019.02.24 |
| *Limnodrilus grandisetosus* | CE1785 | - | KY637016 | Tehang Lake, Central Kalimantan, Indonesia | 113.934 E | 2.029 S | 2005.03.21 |

Continued

| **Species** | **Specimen ID** | **COI** | **ITS2** | **Collection site** | **Longitude** | **Latitude** | **Collection date** |
| --- | --- | --- | --- | --- | --- | --- | --- |
| *Limnodrilus profundicola* | CW0150 | **MT460097** | **MT451912** | Erdaohe River, Heilongjiang, China | 126.458 E | 46.005 N | 2019.07.01 |
| *Limnodrilus profundicola* | CW0151 | **MW888730** | - | Erdaohe River, Heilongjiang, China | 126.458 E | 46.005 N | 2019.07.01 |
| *Limnodrilus profundicola* | CW0152 | **MT460098** | **MT451916** | Erdaohe River, Heilongjiang, China | 126.458 E | 46.005 N | 2019.07.01 |
| *Ilyodrilus templetoni* | CW0056 | **MW888731** | **MW885200** | Taihu Lake, Jiangsu, China | 120.122 E | 31.503 N | 2019.02.23 |
| *Ilyodrilus templetoni* | CW0058 | **MW888732** | - | Taihu Lake, Jiangsu, China | 120.122 E | 31.503 N | 2019.02.23 |
| *Ilyodrilus templetoni* | CE282 | - | KF366654 | Vörtsjärv Limnological Station, Rannu, Estonia | 26.110 E | 58.212 N | 2000.12.01 |
| *Teneridrilus mastix* | CW0031 | **MW888733** | **MW885202** | Taihu Lake, Jiangsu, China | 120.152 E | 31.403 N | 2019.02.23 |
| *Teneridrilus mastix* | CW0032 | **MW888734** | - | Taihu Lake, Jiangsu, China | 120.152 E | 31.403 N | 2019.02.23 |
| *Teneridrilus mastix* | CW0033 | **MW888735** | **MW885204** | Taihu Lake, Jiangsu, China | 120.346 E | 31.953 N | 2019.02.24 |
| *Isochaetides palmatus* | CW0559 | **MW888736** | **MW885205** | Gecuo, Xizang, China | 91.676 E | 28.754 N | 2020.08.30 |
| *Isochaetides palmatus* | CW0560 | **MW888737** | **MW885206** | Gecuo, Xizang, China | 91.676 E | 28.754 N | 2020.08.30 |
| *Isochaetides palmatus* | CW0561 | **MW888738** | - | Gecuo, Xizang, China | 91.676 E | 28.754 N | 2020.08.30 |
| *Tubifex tubifex* | CW0520 | **MW888739** | - | Lalu wetland, Xizang, China | 91.104 E | 29.665 N | 2020.08.23 |
| *Tubifex tubifex* | CW0521 | **MW888740** | **MW885208** | Lalu wetland, Xizang, China | 91.104 E | 29.665 N | 2020.08.23 |
| *Tubifex tubifex* | CW0522 | **MW888741** | - | Lalu wetland, Xizang, China | 91.104 E | 29.665 N | 2020.08.23 |
| *Tubifex tubifex* | T9_116.1 | - | LN810209 | - | - | - | - |
| *Tubifex laxus* | CW0569 | **MW888742** | **MW885209** | Cuomujiri, Xizang, China | 94.421 E | 29.802 N | 2020.08.26 |
| *Tubifex laxus* | CW0570 | **MW888743** | **MW885210** | Cuomujiri, Xizang, China | 94.421 E | 29.802 N | 2020.08.26 |
| *Tubifex laxus* | CW0574 | **MW888744** | - | Cuomujiri, Xizang, China | 94.421 E | 29.802 N | 2020.08.26 |
| *Tubifex laxus* | CW0575 | **MW888745** | - | Cuomujiri, Xizang, China | 94.421 E | 29.802 N | 2020.08.26 |

Continued

| **Species** | **Specimen ID** | **COI** | **ITS2** | **Collection site** | **Longitude** | **Latitude** | **Collection date** |
| --- | --- | --- | --- | --- | --- | --- | --- |
| *Tubifex conicus* | CW0593 | **MW888746** | **MW885211** | Gecuo, Xizang, China | 91.676 E | 28.754 N | 2020.08.30 |
| *Tubifex conicus* | CW0594 | **MW888747** | **MW885212** | Gecuo, Xizang, China | 91.676 E | 28.754 N | 2020.08.30 |
| *Tubifex newaensis* | CE272 | - | KY637047 | Vörtsjärv Limnological Station, Rannu, Estonia | 26.110 E | 58.212 N | 2000.12.01 |
| *Tubifex ignotus* | T6_268 | - | LN810321 | - | - | - | - |
| *Tubifex blanchardi* | CE2044 | - | KY637046 | Paddebeek River, Oost-Vlaanderen, Belgium | 4.05 E | 51.02 N | 2006.09.07 |
| *Potamothrix bedoti* | CE275 | - | KF366653 | Vörtsjärv Limnological Station, Rannu, Estonia | 26.110 E | 58.212 N | 2000.12.01 |
| *Potamothrix moldaviensis* | CE283 | - | KY637042 | Vörtsjärv Limnological Station, Rannu, Estonia | 26.110 E | 58.212 N | 2000.12.01 |
| *Potamothrix hammoniensis* | CE278 | - | KY637041 | Vörtsjärv Limnological Station, Rannu, Estonia | 26.110 E | 58.212 N | 2000.12.01 |
| *Potamothrix bavaricus* | CE570 | - | KY637040 | Man-made freshwater pond, S of  Esperance Municipal Museum, Australia | 121.893 E | 33.860 S | 2003.02.09 |
| *Aulodrilus pluriseta* | CW0184 | **MW889895** | **MW885213** | Donghu Lake, Hubei | 114.352 E | 30.535 N | 2019.08.23 |
| *Aulodrilus pluriseta* | CE281 | - | KY637028 | Vörtsjärv Limnological Station, Rannu, Estonia | 26.110 E | 58.212 N | 2000.12.01 |
| *Aulodrilus pigueti* | CW0185 | **MW889043** | **MW885214** | Donghu Lake, Hubei, China | 114.352 E | 30.535 N | 2019.08.22 |
| *Aulodrilus pigueti* | CW0586 | **MW888748** | **MW885215** | Donghu Lake, Hubei, China | 114.352 E | 30.535 N | 2020.09.25 |
| *Nais pardalis* | CW0175 | **MW888992** | - | Guanlanhe River, Shenzhen, China | 114.037 E | 22.650 N | 2019.08.09 |
| *Nais pardalis* | CW0200 | **MW888749** | - | Shibawan, Jiangsu, China | 120.171 E | 31.535 N | 2019.11.21 |

Continued

| **Species** | **Specimen ID** | **COI** | **ITS2** | **Collection site** | **Longitude** | **Latitude** | **Collection date** |
| --- | --- | --- | --- | --- | --- | --- | --- |
| *Nais pardalis* | CW0202 | **MW888750** | - | Zhushanwan, Jiangsu, China | 120.067 E | 31.414 N | 2019.11.22 |
| *Nais pardalis* | CW0203 | **MW888751** | - | Shibawan, Jiangsu, China | 120.171 E | 31.535 N | 2019.11.23 |
| *Nais pardalis* | CW0481 | - | **MW885216** | Donghu Lake, Hubei, China | 114.352 E | 30.535 N | 2020.08.04 |
| *Nais inflata* | CW0280 | **MW888752** | **MW885217** | Heishuihe River, Yunnan, China | 103.253 E | 25.999 N | 2019.04.16 |
| *Nais bretscheri* | CW0178 | **MW881251** | **MW885218** | Quanjihe River, Qinghai, China | 99.904 E | 37.390 N | 2019.07.23 |
| *Nais bretscheri* | N2_296 | - | LN810237 | - | - | - | - |
| *Nais elinguis* | CW0501 | **MW888753** | **-** | Datonghe River, Qinghai, China | 101.768 E | 36.766 N | 2020.08.14 |
| *Nais elinguis* | CW0502 | **MW888754** | **-** | Datonghe River, Qinghai, China | 101.768 E | 36.766 N | 2020.08.14 |
| *Nais elinguis* | CW0503 | **MW888755** | **MW885219** | Datonghe River, Qinghai, China | 101.768 E | 36.766 N | 2020.08.14 |
| *Nais elinguis* | CE3486 | - | JQ599191 | Experimental biofilter, Manchester Metropolitan Univ, United Kingdom | - | - | - |
| *Nais simplex* | DH21-1 | **MW888756** | **MW885220** | Donghu Lake, Hubei, China | 114.352 E | 30.535 N | 2020.07.29 |
| *Nais communis* | DH23-2 | **MW888757** | **MW885221** | Donghu Lake, Hubei, China | 114.352 E | 30.535 N | 2020.07.29 |
| *Nais communis*/*variabilis* | CE2043 | - | JQ599175 | Lab aquarium with Xenopus frogs, Univ Gothenburg, Sweden | - | - | - |
| *Nais longidentata* | CW0589 | **MW888758** | **MW885222** | Naqu, Xizang, China | 91.740 E | 31.622 N | 2020.09.03 |
| *Nais longidentata* | CW0590 | **MW888759** | **MW885223** | Yejiuqu, Xizang, China | 91.580 E | 28.676 N | 2020.08.30 |
| *Nais badia* | CW0556 | **MW888760** | **MW885224** | Cuomujiri, Xizang, China | 94.416 E | 29.793 N | 2020.08.26 |
| *Nais badia* | CW0558 | **MW888761** | **MW885225** | Cuomujiri, Xizang, China | 94.416 E | 29.793 N | 2020.08.26 |
| *Uncinais uncinata* | CW0541 | **MW888762** | - | Cuomujiri, Xizang, China | 94.421 E | 29.802 N | 2020.08.26 |
| *Uncinais uncinata* | CW0542 | **MW888763** | - | Cuomujiri, Xizang, China | 94.421 E | 29.802 N | 2020.08.26 |
| *Uncinais uncinata* | CW0543 | **MW888764** | **MW885228** | Cuomujiri, Xizang, China | 94.421 E | 29.802 N | 2020.08.26 |
| *Uncinais uncinata* | CE609 | - | KY633376 | Lången Lake, Vårgårda, Sweden | - | - | - |

Continued

| **Species** | **Specimen ID** | **COI** | **ITS2** | **Collection site** | **Longitude** | **Latitude** | **Collection date** |
| --- | --- | --- | --- | --- | --- | --- | --- |
| *Branchiodrilus hortensis* | CW0374 | **MW888765** | **-** | Baoanhu Lake, Hubei, China | 114.730 E | 30.252 N | 2020.06.19 |
| *Branchiodrilus hortensis* | CW0375 | **MW888766** | **-** | Baoanhu Lake, Hubei, China | 114.730 E | 30.252 N | 2020.06.19 |
| *Paranais frici* | CW0279 | **MW888767** | **MW885229** | Heishuihe River, Yunnan, China | 103.253 E | 25.999 N | 2019.04.16 |
| *Paranais frici* | CW0356 | **MW888768** | - | The Yangtze River, Hubei, China | 114.398 E | 30.693 N | 2020.05.15 |
| *Paranais frici* | CE2466 | - | KY633382 | Rappahannock River (brackish), Middlesex Co., VA, USA | - | - | - |
| *Slavina appendiculata* | CW0278 | **MW888769** | **MW885230** | Naolihe River, Heilongjiang, China | 132.651 E | 46.757 N | 2019.10.01 |
| *Slavina appendiculata* | CE207 | - | KY633371 | Lången Lake, Vårgårda, Sweden | - | - | - |
| *Stylaria fossularis* | DH20-1 | **MW888770** | **-** | Donghu Lake, Hubei, China | 114.352 E | 30.535 N | 2020.07.29 |
| *Stylaria fossularis* | DH20-2 | **MW888771** | **MW885232** | Donghu Lake, Hubei, China | 114.352 E | 30.535 N | 2020.07.29 |
| *Stylaria fossularis* | CE1807 | - | KY633374 | Tonle Sap Lake, Cambodia | - | - | - |
| *Chaetogaster limnaei* | DH28-1 | **MW888772** | **MW885233** | Donghu Lake, Hubei, China | 114.352 E | 30.535 N | 2020.07.29 |
| *Chaetogaster limnaei* | DH28-2 | **MW888773** | - | Donghu Lake, Hubei, China | 114.352 E | 30.535 N | 2020.07.29 |
| *Haemonais waldvogeli* | CW0137 | **MW888774** | **MW885234** | Naolihe River, Heilongjiang, China | 132.995 E | 46.813 N | 2019.07.05 |
| *Haemonais waldvogeli* | CW0211 | **MW888775** | - | Liyang, Jiangsu, China | 119.501 E | 31.274 N | 2019.11.17 |
| *Haemonais waldvogeli* | CW0212 | **MW888776** | - | Wulihu Lake, Jiangsu, China | 120.248 E | 31.519 N | 2019.11.22 |
| *Haemonais waldvogeli* | CW0213 | **MW888777** | - | Wulihu Lake, Jiangsu, China | 120.248 E | 31.519 N | 2019.11.22 |
| *Aulophorus furcatus* | CW0209 | **MW888778** | - | Wulihu Lake, Jiangsu, China | 120.248 E | 31.519 N | 2019.11.24 |
| *Aulophorus furcatus* | CW0210 | **MW888779** | - | Wulihu Lake, Jiangsu, China | 120.248 E | 31.519 N | 2019.11.25 |
| *Dero dorsalis* | CW0155 | **MW889891** | **MW885235** | Majiagou, Heilongjiang, China | 126.633 E | 45.722 N | 2019.06.28 |
| *Dero dorsalis* | CW0156 | **MW889892** | **MW885236** | Majiagou, Heilongjiang, China | 126.633 E | 45.722 N | 2019.06.28 |

Continued

| **Species** | **Specimen ID** | **COI** | **ITS2** | **Collection site** | **Longitude** | **Latitude** | **Collection date** |
| --- | --- | --- | --- | --- | --- | --- | --- |
| *Dero obtusa* | DH10-8 | **MW888780** | **MW885237** | Donghu Lake, Hubei, China | 114.352 E | 30.535 N | 2020.07.29 |
| *Dero obtusa* | DH10-9 | **MW888781** | **MW885238** | Donghu Lake, Hubei, China | 114.352 E | 30.535 N | 2020.07.29 |
| *Dero digitata* | CW0272 | **MW888782** | - | Naolihe River, Heilongjiang, China | 132.651 E | 46.757 N | 2019.10.01 |
| *Dero digitata* | CE489 | - | KY633381 | Lången Lake, Vårgårda, Sweden, China | - | - | - |
| *Bothrioneurum vejdovskyanum* | CW0603 | **MW888783** | - | Lalu wetland, Xizang, China | 91.104 E | 29.665 N | 2020.08.24 |
| *Bothrioneurum vejdovskyanum* | CW0604 | **MW888784** | **MW885240** | Lalu wetland, Xizang, China | 91.104 E | 29.665 N | 2020.08.24 |
| *Bothrioneurum vejdovskyanum* | CE286 | - | KY637030 | Vörtsjärv Limnological Station, Rannu, Estonia | 26.110 E | 58.212 N | 2000.12.01 |
| *Rhyacodrilus sinicus* | CW0564 | **MW888785** | **MW885241** | Lulang, Xizang, China | 94.607 E | 29.598 N | 2020.08.28 |
| *Rhyacodrilus sinicus* | CW0565 | **MW888786** | **MW885242** | Lulang, Xizang, China | 94.607 E | 29.598 N | 2020.08.28 |
| *Rhyacodrilus sinicus* | CW0566 | **MW888787** | **MW885243** | Lulang, Xizang, China | 94.607 E | 29.598 N | 2020.08.28 |
| *Rhyacodrilus subterraneus* | CE10572 | - | KF267995 | Spring, Willesleigh Farm, Devon, Great Britain | - | - | 2010.01 |
| *Rhyacodrilus coccineus* | CE623 | - | KF267996 | Stream, Alingsås, Sweden | - | - | 2003.07 |
| *Rhyacodrilus falciformis* | CE4563 | - | KF267989 | Stream, Frösön, Östersund, Sweden | - | - | 2008.07 |
| *Lumbriculus variegatus* | CW0259 | **MW888788** | **MW885246** | Naolihe River, Heilongjiang, China | 132.892 E | 46.788 N | 2019.10.01 |
| *Lumbriculus variegatus* | CW0263 | **MW888789** | **MW885247** | Naolihe River, Heilongjiang, China | 132.892 E | 46.788 N | 2019.10.01 |
| *Lumbriculus variegatus* | CW0264 | **MW888790** | **MW885248** | Naolihe River, Heilongjiang, China | 132.892 E | 46.788 N | 2019.10.01 |
| *Lumbriculus variegatus* | CE1993 | - | FJ639320 | Lången Lake, near Alingsås, Sweden | - | - | - |
